# Effect of Stress Ulcers Prophylaxis, Sedative and Statin on Ventilator-Associated Pneumonia: A Retrospective Analysis Based on MIMIC Database

**DOI:** 10.3389/fphar.2022.921422

**Published:** 2022-06-20

**Authors:** Xuetao Kong, Yaozhou Wu, Bingqin Wen, Dongmei Meng, Li Wei, Pengjiu Yu

**Affiliations:** ^1^ National Clinical Research Center for Respiratory Disease, State Key Laboratory of Respiratory Disease, Guangzhou Institute of Respiratory Health, The First Affiliated Hospital of Guangzhou Medical University, Guangzhou, China; ^2^ Department of Pharmacy, The First Affiliated Hospital of Guangzhou Medical University, Guangzhou, China

**Keywords:** mechanical ventilation, ventilator-associated pneumonia, MIMIC IV database, stress ulcers prophylaxis, sedative, statin

## Abstract

**Background:** The use of MV can easily lead to VAP especially in ICU patients. SUP, sedatives, statin and insulin have been proved to prevent VAP and improve the prognosis of patients. Our aim was to analyze the effects of SUP, sedative, statin, and insulin on patients with MV.

**Methods:** The occurrence of VAP and death in MV patients and VAP patients were explored by multivariate logistic regression and Cox regression to analyze analyses.

**Results:** Totally, 5277 cases who received MV in ICU from MIMIC IV database were included. There were 826 (15.7%) cases in VAP-group and 4451 (84.3%) cases in non-VAP group and there were 1914 (36.3%) cases in hospital mortalities altogether. No protective effect of drugs on VAP was found in MV patients. The risk of death was 1.43 times higher in MV patients taking midazolam than in propofol (aHR = 1.43 95% CI: 1.04,1.97). No protective effect of drugs on death was found in VAP patients.

**Conclusion:** Compared with midazolam, propofol is more recommended as sedation regimen in ICU patients with MV. Further high-quality studies are needed to confirm this finding.

## Background

Ventilator-associated pneumonia (VAP) is the second most common nosocomial infection in ICU, with an incidence rate ranging from 8%–28% and a mortality rate ranging from 8%–76% (Vincent et al., 1995; Rello et al., 2002), and patients in intensive care unit (ICU) with VAP are 1–9 times more likely to die. If the patients admitted to the intensive care unit (ICU) are complicated with VAP, the mortality rate will increase by 1–9 times (Celik et al., 2012; Yamada et al., 2012). The presence of a tracheal tube is the most important risk factor for the development of VAP. The existence of the catheter can interfere with the normal protective upper airway reflexes and inhibits effective coughing, leading to the inhalation of contaminated oropharyngeal secretions and gradually developing into pulmonary infection lesions (Adair et al., 1999; Zolfaghari and Wyncoll, 2011; Hunter, 2012). Therefore, the chance of pneumonia increases when the duration of MV is prolonged. The methods of treating VAP in ICU mainly include antibiotic therapy, sputum suction and nutritional support, but most patients have a poor prognosis (Luo et al., 2021). Since no effective drugs have been found to treat VAP, it has become the focus of attention to find drugs that can prevent the occurrence of VAP or improve the prognosis of patients with VAP.

Oversedation can cause serious adverse reactions to patients, such as cardiorespiratory depression, decreased gastrointestinal motility, immunosuppression and unnecessary prolongation of MV, thus, indirectly increasing the risk of infection. Undersedation may result in hypertension, tachycardia and severe discomfort (Garrett, 2016). Therefore, choosing an appropriate sedation regimen is crucial. Propofol, dexmedetomidine and midazolam are common sedative drugs used in clinical practice. A preliminary study showed that dexmedetomidine reduced the incidence of coma and insanity in patients and shortened the duration of MV compared to propofol or midazolam (Pandharipande et al., 2007). Another meta-analysis found that propofol improved clinical outcomes in the ICU and reduced time to extubation in critically ill patients compared with midazolam (Garcia et al., 2021). The results of the above studies have been inconsistently described and no definitive conclusions have been made regarding the effect of the three sedation regimens on patient sedation and prognosis.

ICU patients are at risk for upper gastrointestinal stress ulcer bleeding, and patients requiring long-term MV are at higher risk (Peura, 1987). Therefore, stress ulcer prevention (SUP) is generally considered to be the standard of care in the ICU (Krag et al., 2013). SUP drugs usually include proton pump inhibitors (PPI), Histamine2-receptor antagonist (H2RA) and sucralfate. A previous study compared the efficacy of ranitidine and sucralfate in patients receiving MV over 48 h. The investigators found a significant decrease in the rate of clinically significant bleeding for patients receiving ranitidine, accompanied by a nonsignificant increase in VAP (Cook et al., 1998). A randomized, open-label, crossover clinical trial reported a higher mortality rate in the PPI group (18.3%) than in the H2RA group, although the difference was not statistically significant (Young et al., 2020). The potential benefits and harms of PPI, H2RA and sucralfate for the prevention of stress ulcers in critically ill patients still require further analysis.

In addition to their proven ability to regulate blood lipids, statins have been shown to have various other pharmacological effects on independent cholesterol pathways, such as antithrombotic and anti-inflammatory effects (Mortensen et al., 2005; van de Garde et al., 2006; Chalmers et al., 2008). Based on this principle, many studies have linked them to VAP, aiming to explore the benefits of statins in ICU patients. A previously published meta-analysis of cohort studies showed that preadmission statin use is associated with beneficial outcomes in critically ill patients, including lower short-term mortality and less use of MV (Yu et al., 2021a). In contrast, another study concluded that in adults with suspected VAP, adjunctive simvastatin therapy compared with placebo did not improve day-28 survival, and VAP patients cannot benefit from it (Papazian et al., 2013a). There are conflicting results regarding the benefit of pleiotropic effects of statins in ICU patients.

Patients admitted to the ICU often have hyperglycemia and insulin resistance in stressful situations even in the absence of a history of diabetes, and significant blood glucose elevations are associated with a worsening prognosis in critically ill patients, mainly including stroke, myocardial infarction, head trauma and postoperative wound infection (Krinsley, 2004). Two previous reports by Van den Berghe stated that intensive insulin therapy (IIT) reduced patient mortality and morbidity in surgical ICU patients, and decreased the risk of death in 767 patients with hospital stay ≥3 days (van den Berghe et al., 2001; Van den Berghe et al., 2006). In contrast, another recently published study showed that intensive insulin therapy was not associated with improved survival in patients in medical and surgical ICUs and was associated with an increased incidence of hypoglycemia (Arabi et al., 2008). Despite the above disputes and concerns, people still tend to make strict blood glucose control in critically ill patients a major treatment goal.

Based on previous studies, we found that the use of sedative drugs, SUP, statins and insulin may be candidates for the prevention and treatment of VAP patients, but the application of drugs has been controversial due to inconsistent results of the research. Therefore, we conducted this retrospective study to verify the effects of the above drugs on patients in ICU.

## Materials and Methods

### Study Design and Participants

Cross-sectional research was conducted in this study. The data was sourced from the MIMIC IV database from 2008 to 2019 which was downloaded from (https://mimic.physionet.org/) (Johnson et al., 2021). The database contains more than 40000 ICU patient medical data from Beth Israel Deaconess Medical Center. We gained access to the MIMIC IV database files upon completion of the required training (COLLABORATIVE INSTITUTIONAL TRAINING INITIATIVE). (Record ID: 47402444).

Eligible participants were age ≥18 years and the duration of MV ≥ 48 h. Patients with multiple mechanical ventilation at different ICU admissions were excluded. To analyze the risk factors of VAP, all patients will be divided into VAP group and non-VAP group and drugs were taken as the independent variable. Because all patient data was de-identified, informed consent was waived.

### Data Collection

Baseline clinical data were extracted from the first admission including age (year), sex, ethnicity (American Indian/Alaska National, Asian, black/African American, Hispanic/Latino, white), simplified acute physiology score II (SAPSII), Charlson comorbidity Index (CCI), clinical disease classification (respiratory, circulation, CNS, liver, renal, diabetes, trauma, other). Leukocyte count, INR, and lactate were extracted from the first day of MV. We extracted bacterial infection [*Acinetobacter* baumannii, *Pseudomonas aeruginosa*, *Klebsiella pneumoniae*, *Escherichia coli*, methicillin resistant Staphylococcus aureus (MRSA), Stenotrophomonas *maltophilia*]. And drugs (antiplatelet, insulin, sedative, statin, PPI, H2, sucralfate) during MV. We also extracted the length of hospital stay (day), ICU duration (day), and ventilation duration (day). The clinical classification of diseases is based on clinical classifications software suggested by the agency for healthcare research and quality (AHRQ) (Cowen et al., 1998).

### Outcomes

The primary outcomes were incidence of VAP and VAP patients’ mortalities and the secondary outcome was MV patients’ mortalities. The definition of VAP is based on the International Classification of Diseases 9th edition and International Classification of Diseases 10th edition (ICD-9: 4957 and 99731; ICD-10: J95851) (World, 2004). The observation period began on the date MV of started and it end at the date of discharge or in-hospital death of the patient.

### Statistical Analysis

Wilcoxon rank sum test was for non-normally distributed data. We used Chi-square and Fisher’s exact test (when the expected value of data was lower than 5) for categorical variables. Several descriptive statistics, including medians and interquartile ranges, are presented. We select confounder variables for multivariable analysis if they had a *p* ≤ 0.1 on univariate analysis. Multivariable logistic regression was used to analyze the VAP independent risk factor and odds ratio (OR), 95% confidence intervals (CI), and p-value were presented. The Cox regression model was used to analyze the factor affecting MV patient survival and VAP patient survival. Statistical analyses were performed in R version 4.1.2.

## Results

### Baseline Characteristics

**FIGURE 1:**
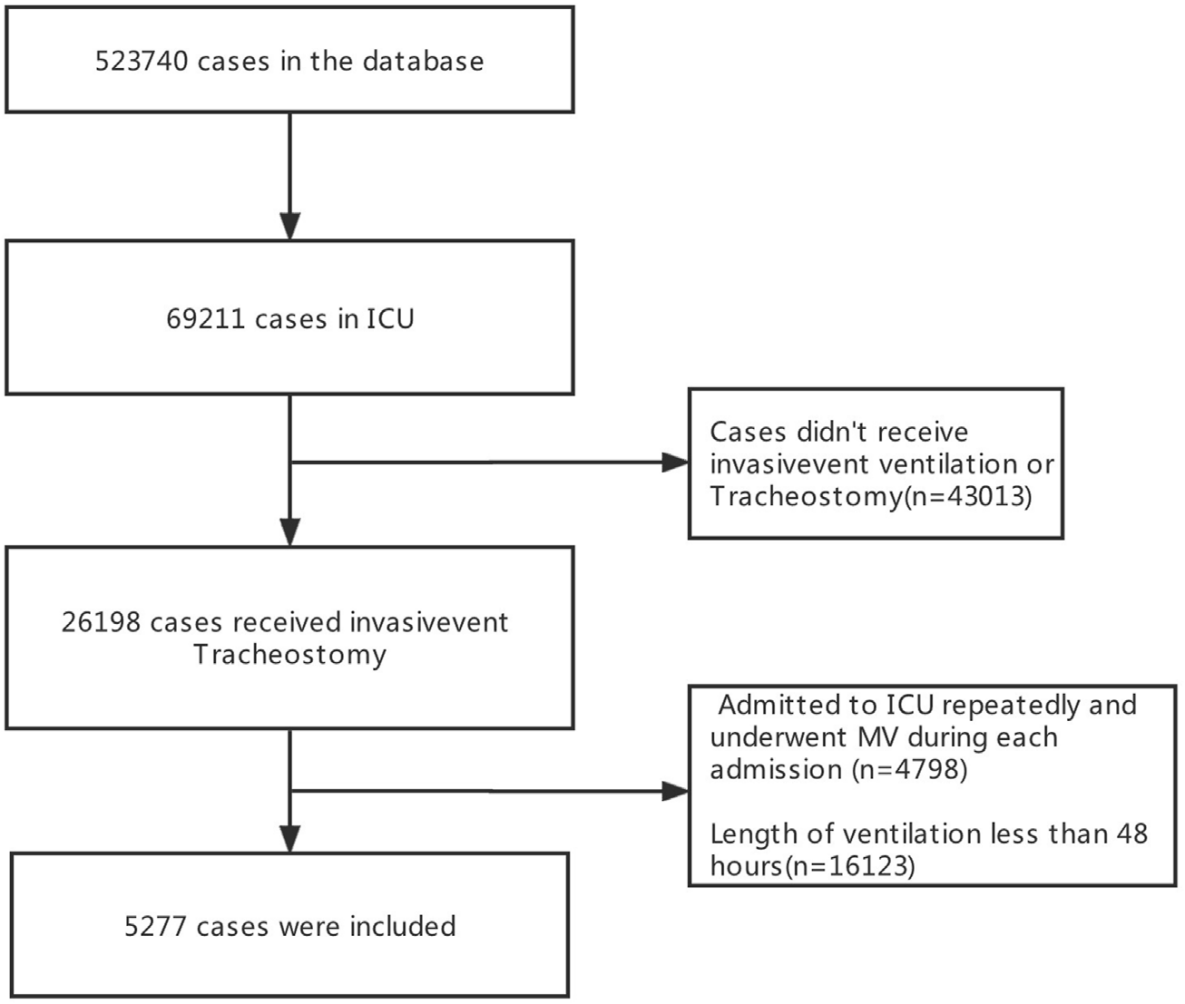
Flowchart of screening phases.

23566 patients were screened in the database and 5277 patients were finally included in the present study (Figure 1). There were 826 (15.7%) patients with VAP and 4451 (84.3%) patients were non-VAP. Among all patients, the median age was 65.4years, 3018 (57.2%) were male, 3179 (60.2%) were White, 794 (15.0%) were diagnosed with respiratory diseases, the median stays in ICU were 8.83 days, and 1914 (36.3%) died in hospital. (Table 1).

**TABLE 1:** Baseline characteristics of mechanical ventilation cases. (n = 5277).

	VAP group (*N* = 826)	Non-VAP group (*N* = 4451)	Overall (*N* = 5277)	*p*-value
**Age**				
Median [min, max]	65.1 [19.6, 96.9]	65.5 [18.0, 98.7]	65.4 [18.0, 98.7]	0.468
**Gender**				
Male	512 (62.0%)	2506 (56.3%)	3018 (57.2%)	**0.003**
Female	314 (38.0%)	1945 (43.7%)	2259 (42.8%)	
**Ethnicity**				
American Indian/Alaska native	2 (0.2%)	12 (0.3%)	14 (0.3%)	0.999
Asian	24 (2.9%)	132 (3.0%)	156 (3.0%)	
Black/African American	89 (10.8%)	491 (11.0%)	580 (11.0%)	
Hispanic/Latino	27 (3.3%)	156 (3.5%)	183 (3.5%)	
White	481 (58.2%)	2698 (60.6%)	3179 (60.2%)	
Missing	203 (24.6%)	962 (21.6%)	1165 (22.1%)	
**SAPS II**				
Median [min, max]	42.0 [6.00, 107]	43.0 [6.00, 107]	43.0 [6.00, 107]	**0.014**
**SOFA**				
Median [min, max]	9.00 [0, 21.0]	9.00 [0, 23.0]	9.00 [0, 23.0]	0.246
**CCI**				
Median [min, max]	6.00 [0, 15.0]	6.00 [0, 19.0]	6.00 [0, 19.0]	0.084
**Diagnoses**				
Respiratory	126 (15.3%)	668 (15.0%)	794 (15.0%)	0.762
CNS	20 (2.4%)	108 (2.4%)	128 (2.4%)	
Liver	10 (1.2%)	71 (1.6%)	81 (1.5%)	
Renal	7 (0.8%)	46 (1.0%)	53 (1.0%)	
Diabetes	4 (0.5%)	22 (0.5%)	26 (0.5%)	
Trauma	57 (6.9%)	254 (5.7%)	311 (5.9%)	
Other	468 (56.7%)	2654 (59.6%)	3122 (59.2%)	
Missing	134 (16.2%)	628 (14.1%)	762 (14.4%)	
**Acinetobacter baumannii**				
N	799 (96.7%)	4412 (99.1%)	5211 (98.7%)	**<0.001**
Y	27 (3.3%)	39 (0.9%)	66 (1.3%)	
**Pseudomonas aeruginosa**				
N	701 (84.9%)	4168 (93.6%)	4869 (92.3%)	**<0.001**
Y	125 (15.1%)	283 (6.4%)	408 (7.7%)	
**Klebsiella pneumoniae**				
N	741 (89.7%)	4234 (95.1%)	4975 (94.3%)	**<0.001**
Y	85 (10.3%)	217 (4.9%)	302 (5.7%)	
**Escherichia coli**				
N	739 (89.5%)	4098 (92.1%)	4837 (91.7%)	**0.016**
Y	87 (10.5%)	353 (7.9%)	440 (8.3%)	
**MRSA**				
N	769 (93.1%)	4187 (94.1%)	4956 (93.9%)	0.322
Y	57 (6.9%)	264 (5.9%)	321 (6.1%)	
**Stenotrophomonas maltophilia**				
N	777 (94.1%)	4348 (97.7%)	5125 (97.1%)	<0.001
Y	49 (5.9%)	103 (2.3%)	152 (2.9%)	
**WBC**				
Median [min, max]	9.85 [0.100, 54.5]	9.80 [0.100, 208]	9.80 [0.100, 208]	0.522
Missing	0 (0%)	6 (0.1%)	6 (0.1%)	
**INR**				
Median [min, max]	1.30 [0.900, 27.4]	1.30 [0.800, 15.2]	1.30 [0.800, 27.4]	0.439
Missing	4 (0.5%)	60 (1.3%)	64 (1.2%)	
**Lactate**				
Median [min, max]	1.50 [0.300, 21.6]	1.60 [0, 28.2]	1.60 [0, 28.2]	**<0.001**
Missing	33 (4.0%)	210 (4.7%)	243 (4.6%)	
**SUP**				
PPI	130 (15.7%)	714 (16.0%)	844 (16.0%)	**0.015**
H2RA	135 (16.3%)	646 (14.5%)	781 (14.8%)	
PPI or Sucralfate	5 (0.6%)	20 (0.4%)	25 (0.5%)	
H2 or Sucralfate	0 (0%)	2 (0.0%)	2 (0.0%)	
PPI or H2RA	68 (8.2%)	203 (4.6%)	271 (5.1%)	
PPI, H2RA or Sucralfate	0 (0%)	1 (0.0%)	1 (0.0%)	
Missing	488 (59.1%)	2865 (64.4%)	3353 (63.5%)	
**Sedative**				
Propofol	106 (12.8%)	649 (14.6%)	755 (14.3%)	**<0.001**
N	507 (61.4%)	2977 (66.9%)	3484 (66.0%)	
Dexmedetomidine	7 (0.8%)	26 (0.6%)	33 (0.6%)	
Midazolam	19 (2.3%)	67 (1.5%)	86 (1.6%)	
Dexmedetomidine or Propofol	86 (10.4%)	335 (7.5%)	421 (8.0%)	
Midazolam or Propofol	46 (5.6%)	247 (5.5%)	293 (5.6%)	
Dexmedetomidine, Midazolam or Propofol	55 (6.7%)	150 (3.4%)	205 (3.9%)	
**Statin**				
N	701 (84.9%)	3930 (88.3%)	4631 (87.8%)	**0.007**
Y	125 (15.1%)	521 (11.7%)	646 (12.2%)	
**Insulin**				
N	599 (72.5%)	3446 (77.4%)	4045 (76.7%)	**0.003**
Y	227 (27.5%)	1005 (22.6%)	1232 (23.3%)	
**Antibiotic**				
N	18 (2.2%)	263 (5.9%)	281 (5.3%)	**<0.001**
Single antibiotic	35 (4.2%)	516 (11.6%)	551 (10.4%)	
Combined antibiotics	773 (93.6%)	3672 (82.5%)	4445 (84.2%)	
**Vasopressor**				
N	596 (72.2%)	3097 (69.6%)	3693 (70.0%)	0.149
Y	230 (27.8%)	1354 (30.4%)	1584 (30.0%)	
**Length of Ventilation (day)**				
Median [min, max]	5.68 [2.00, 52.5]	3.70 [2.00, 85.3]	3.92 [2.00, 85.3]	**<0.001**
**Length of ICU stays (day)**				
Median [min, max]	13.5 [2.33, 79.0]	8.11 [2.10, 99.6]	8.83 [2.10, 99.6]	**<0.001**
**In-hospital mortalities**				
N	574 (69.5%)	2789 (62.7%)	3363 (63.7%)	**<0.001**
Y	252 (30.5%)	1662 (37.3%)	1914 (36.3%)	

SAPS II, Simplified acute physiology score; SOFA, Sequential Organ Failure Assessment; CCI, Charlson comorbidity Index; CNS, Central nervous system; MRSA, Methicillin-resistant Staphylococcus; WBC, White blood cell; INR, International Normalized Ratio; PPI, Proton pump inhibitors; H2RA, Histamine2-receptor antagonist; SUP, stress ulcer prevention; VAP, Ventilator-associated pneumonia; N, No; Y, Yes.

### Comparison of Characteristics Between VAP Group and Non-VAP Group

The gender of the VAP group and the non-VAP group was different, the SAPS II score of the VAP group was lower than that of the non-VAP group (42.0 vs. 43.0, *p* < 0.014). VAP Patients detected *Acinetobacter* baumannii, *Pseudomonas aeruginosa*, *Klebsiella* pneumonia, *Escherichia coli*, and Stenotrophomonas *maltophilia* more than non-VAP patients (3.3 *vs*. 0.9%, *p* < 0.001; 15.1 *vs*. 6.4%, *p* < 0.001; 10.3 *vs*. 4.9%, *p* < 0.001; 10.5 *vs*. 7.9%, *p* = 0.016; 5.9 *vs*. 2.3%, *p* < 0.001). The lactate of the VAP group was lower than that of the non-VAP group (1.50 *vs*. 1.60, *p* < 0.001). The use of sedatives and stress ulcer prophylaxis was different between the VAP group and the non-VAP group. More statins users in VAP group (15.1 *vs*. 11.7%, *p* = 0.007). More insulin users in VAP group (27.5 *vs*. 22.6%, *p* = 0.007). The use of antibiotics was different between the VAP group and the non-VAP group. The length of ventilation of VAP patients was longer than that of non-VAP patients (5.68 *vs*. 3.70, *p* < 0.001). The length of ICU stays of VAP patients was longer than that of non-VAP patients (13.5 *vs*. 8.11, *p* < 0.001). And the in-hospital mortalities in VAP group was lower (30.5 *vs* 37.3%, *p* < 0.001). (Table 1).

### Influence of Drugs on VAP in MV Patients

After adjustment for confounders, there was no statistically significant difference in VAP risk between PPI and H2RA (aOR = 1.11 95% CI: 0.83, 1.47). There was also no statistically significant difference in the risk of VAP between propofol, dexmedetomidine and midazolam (aOR = 1.61, 95% CI: 0.65,4.02; aOR = 1.54, 95% CI: 0.81,2.9). Taking statins did not significantly increase risk factor for VAP (aOR = 1.22 95% CI: 0.92, 1.62). Insulin also did not significantly reduce the risk of VAP (aOR = 0.94 95% CI: 0.71,1.25). (Table 2).

**TABLE 2:** Logistic regression analysis of VAP in MV patients. (n=5277)

Covariate	aOR (95%CI)	*p*-value
**Gender**		**0.023**
Male	reference	
Female	0.73 (0.56,0.96)	
**SAPS II**	1.00 (0.99,1.01)	0.535
**CCI**	0.98 (0.94,1.03)	0.528
**Acinetobacter baumannii**		0.070
N	reference	
Y	2.48 (0.93,6.61)	
**Pseudomonas aeruginosa**		**0.008**
N	reference	
Y	1.79 (1.17,2.76)	
**Klebsiella pneumoniae**		**0.002**
N	reference	
Y	2.03 (1.29,3.18)	
**Escherichia coli**		0.377
N	reference	
Y	1.23 (0.78,1.92)	
**Stenotrophomonas maltophilia**		0.719
N	reference	
Y	1.14 (0.56,2.31)	
**WBC**	1.00 (0.98,1.01)	0.800
**Lactate**	0.93 (0.88,0.99)	**0.017**
**SUP**		0.880
PPI	reference	
H2RA	1.11 (0.83,1.47)	0.487
PPI or Sucralfate	1.54 (0.54,4.37)	0.414
H2 or Sucralfate	NA	NA
PPI or H2RA	1.23 (0.85,1.8)	0.275
PPI, H2RA or Sucralfate	NA	NA
**Sedative**		0.210
Propofol	reference	
N	0.94 (0.54,1.65)	0.835
Dexmedetomidine	1.61 (0.65,4.02)	0.304
Midazolam	1.54 (0.81,2.9)	0.190
Dexmedetomidine or Propofol	1.26 (0.9,1.76)	0.187
Midazolam or Propofol	0.84 (0.56,1.27)	0.419
Dexmedetomidine, Midazolam or Propofol	1.42 (0.93,2.16)	0.108
**Statin**		0.161
N	reference	
Y	1.22 (0.92,1.62)	
**Insulin**		0.684
N	reference	
Y	0.94 (0.71,1.25)	
**Antibiotic**		**0.005**
N	reference	
Single	1.14 (0.38,3.4)	0.817
Combined	2.59 (1.01,6.62)	**0.047**
**Length of Ventilation (day)**	1.01 (0.97,1.04)	0.720
**Length of ICU stays (day)**	1.07 (1.04,1.09)	*<* **0.001**
**In-hospital mortalities**		0.314
N	reference	
Y	0.86 (0.64,1.16)	

aOR (95%CI), adjusted Odds ratio (95% confidence interval).

### Influence of Drugs on the Risk of Death in MV Patients

There were 1914 (36.3%) in-hospital mortalities among mechanical ventilation patients. After adjusting for confounding factors (Supplementary Table S1), the results showed no statistically significant difference in VAP risk between PPI and H2RA (aOR = 1.11 95% CI: 0.83, 1.47). There was also no statistically significant difference in the risk of VAP between propofol, dexmedetomidine and midazolam (aOR = 1.61, 95% CI: 0.65,4.02; aOR = 1.54, 95% CI: 0.81,2.9). Taking statins did not significantly increase risk factor for VAP (aOR = 1.22 95% CI: 0.92, 1.62). Insulin also did not significantly reduce the risk of VAP (aOR = 0.94 95% CI: 0.71,1.25). (Table 3).

**TABLE 3:** Survival analysis of patients with mechanical ventilation.

Covariate	aHR(95%CI)	*p*-value
**Age**	1.01 (1.00,1.01)	**0.024**
**Gender**		0.147
** **Male	reference	
** **Female	1.12 (0.96,1.30)	
**SAPS II**	0.99 (0.99,1.00)	**0.0281**
**SOFA**	1.01 (0.99,1.04)	0.210
**CCI**	1.07 (1.04,1.10)	***<*0.001**
**Diagnoses**		0.580
Respiratory	reference	
CNS	0.71 (0.42,1.22)	0.218
Liver	0.80 (0.47,1.36)	0.412
Renal	0.90 (0.51,1.57)	0.706
Diabetes	0.58 (0.18,1.86)	0.357
Trauma	0.94 (0.70,1.27)	0.705
Other	0.82 (0.65,1.04)	0.099
**Pseudomonas aeruginosa**		0.202
N	reference	
Y	0.81 (0.59,1.12)	
**Stenotrophomonas maltophilia**		0.181
N	reference	
Y	0.75 (0.50,1.14)	
**WBC**	1.02 (1.01,1.02)	***<*0.001**
**INR**	1.10 (1.03,1.17)	**0.006**
**Lactate**	1.08 (1.06,1.10)	***<*0.001**
**SUP**		0.256
PPI	reference	
H2RA	1.17 (0.99,1.38)	0.0712
PPI or Sucralfate	0.93 (0.51,1.70)	0.811
H2 or Sucralfate	2.25 (0.55,9.15)	0.256
PPI or H2RA	1.25 (1.01,1.55)	**0.043**
PPI, H2RA or Sucralfate	NA	NA
**Sedative**		***<*0.001**
Propofol	reference	
N	1.99 (1.53,2.58)	***<*0.001**
Dexmedetomidine	0.75 (0.41,1.40)	0.370
Midazolam	1.43 (1.04,1.97)	**0.026**
Dexmedetomidine or Propofol	0.66 (0.53,0.81)	***<*0.001**
Midazolam or Propofol	1.13 (0.91,1.39)	0.272
Dexmedetomidine, Midazolam or Propofol	0.56 (0.42,0.76)	***<*0.001**
**Statin**		0.305
N	reference	
Y	1.09 (0.92,1.29)	
**Insulin**		0.098
N	reference	
Y	0.87 (0.74,1.03)	
**Antibiotic**		**0.044**
N	reference	
Single	0.53 (0.30,0.93)	**0.027**
Combined	0.8 0 (0.50,1.26)	0.332
**Vasopressor**		***<*0.001**
N	reference	
Y	1.91 (1.61,2.26)	
**Length of Ventilation (day)**	1.15 (1.12,1.18)	***<*0.001**
**VAP**		0.927
N	reference	
Y	0.99 (0.80,1.22)	
**Length of ICU stays (day)**	0.85 (0.83,0.86)	***<*0.001**

aHR (95%CI), adjusted Hazard Ratio (95% confidence interval); p-value < 0.05 is highlighted with bold values.

### Effect of Drugs on Death in VAP Patients

There were 252 (30.5%) in-hospital mortalities among ventilator-associated pneumonia patients. After adjusting for confounding factors (Supplementary Table S2), compared with VAP patients taking PPI, patients taking H2RA do not have a greater risk of death (aHR = 1.35 95% CI: 0.81,2.25). There was also no significant reduction in the risk of death after taking dexmedetomidine (aHR = 0.88 95% CI: 0.19,4.07). Taking midazolam also did not increase the risk of death in VAP patients (aHR = 1.69 95% CI: 0.70,4.06). Taking statin and insulin did not increase the risk of death in VAP patients (aHR = 1.00 95% CI: 0.64, 1.56; aHR = 1.20 95% CI: 0.71, 2.02) (Table 4).

**TABLE 4:** Survival analysis of VAP patients. (*n* = 826).

Covariate	aHR (95%CI)	*p*-value
**Age**	1.01 (0.99,1.03)	0.259
**SAPS II**	1.00 (0.98,1.02)	0.718
**SOFA**	1.01 (0.94,1.08)	0.759
**Diagnoses**		0.077
Respiratory	reference	
CNS	0.96 (0.21,4.45)	0.963
Liver	2.21 (0.57,8.51)	0.251
Renal	1.50 (0.28,8.14)	0.637
Diabetes	NA	NA
Trauma	2.32 (1.12,4.79)	**0.023**
Other	0.98 (0.53,1.81)	0.951
**WBC**	1.06 (1.04,1.09)	***<*0.001**
**INR**	1.10 (0.82,1.47)	0.543
**Lactate**	1.04 (0.95,1.14)	0.420
**CCI**	1.03 (0.95,1.11)	0.598
**SUP**		0.320
PPI	reference	
H2RA	1.35 (0.81,2.25)	0.242
PPI or Sucralfate	3.45 (0.75,15.78)	0.111
PPI or H2RA	1.30 (0.76,2.24)	0.339
**Sedative**		**0.027**
Propofol	reference	
N	2.44 (1.13,5.28)	**0.023**
Dexmedetomidine	0.88 (0.19,4.07)	0.874
Midazolam	1.69 (0.70,4.06)	0.244
Dexmedetomidine or Propofol	0.61 (0.35,1.08)	0.093
Midazolam or Propofol	0.76 (0.41,1.39)	0.369
Dexmedetomidine, Midazolam or Propofol	0.72 (0.37,1.38)	0.324
**Statin**		0.987
N	reference	
Y	1.00 (0.64,1.56)	
**Insulin**		0.506
N	reference	
Y	1.20 (0.71,2.02)	
**Antibiotic**		0.410
N	reference	
Single	1.59 (0.13,18.74)	0.713
Combined	3.01 (0.39,23.45)	0.292
**Vasopressor**		0.116
N	reference	
Y	1.45 (0.91,2.31)	
**Length of Ventilation (day)**	0.96 (0.93,0.99)	**0.012**

*p*-value < 0.05 is highlighted with bold values.

## Discussion

The present study retrospectively analyzed the underlying therapeutic effects of sedative drugs, SUP, statins, and insulin in patients with MV by using the MIMIC database. Our results found that patients with MV benefited more with propofol than with midazolam.

This study analyzed the effects of different SUP regimens in patients with MV. The results showed that patients with MV using sucralfate or H2RA had a similar risk of death to those with MV using PPI, and there were no differences in the occurrence of VAP and the prognosis of VAP patients. A previous meta-analysis including randomized clinical trials showed that PPI may be more effective for preventing upper gastrointestinal bleeding than H2RA, but had no differences between drugs in the risk of pneumonia, death, or ICU length of stay. This result has some limitations, such as insufficient data, differences between lower and higher quality trials, methodological limitations, and possible publication bias (Alhazzani et al., 2013). Another study concluded that the incidence of nosocomial pneumonia were not different between patients using PPIs and those using H2RA. However, for prevention of stress-related mucosal diseases, the rate of clinically important bleeding decreased significantly in patients using PPI (Pongprasobchai et al., 2009). All of the above reports are consistent with our findings. For MV patients who need to receive SUP, since there is no clear conclusion to prove the difference between H2RA and PPI, when using acid suppressants, we should give priority to the characteristics of the patient and disease, and choose a more appropriate SUP regimen. In addition, previous studies have shown that sucralfate did not affect the gastric pH of patients, and did not increase the risk of bacteria infection (Kappstein et al., 1991; Sun et al., 2019). Thus, many studies have illuminated that sucralfate should be the preferred option for SUP compared to PPI and H2RA. However, in our study, due to few patients used sucralfate, the corresponding p-values could not be calculated. We hope that a prospective cohort study with a larger sample size will confirm this conclusion in the future.

Our study also compared the effects of different sedation regimens. The results showed that patients in the midazolam group had a higher risk of death than those in the propofol group, but we did not find differences between several sedation regimens in terms of the occurrence of VAP and the prognosis of patients with VAP. The 2013 Pain, Agitation, and Delirium (PAD) guidelines stated that non-benzodiazepine is better choice than benzodiazepines for mechanically ventilated adult patients sinceof the former improved length of stay, duration of MV, 90-day mortality and psychological dysfunction in ICU patients (Barr et al., 2013). Another study showed that the 28-day mortality rate of patients treated with midazolam was 30.8%, and the propofol group was 25.5%, the adjusted odds ratio (OR) value was 1.421 [95% confidence interval (CI): 1.118–1.806, *p* < 0.001], with a significantly lower mortality rate in the propofol group (Sun et al., 2022). Many previous studies do not support the choice of midazolam as a sedation regimen in patients with MV, mainly considering that midazolam is easy to accumulate in patients’ tissues, resulting in prolonged metabolism and elimination time (Spina and Ensom, 2007). On the other hand, propofol has the advantages of quick effect, rapid distribution and metabolism, and is less likely to produce sequelae, which make it an ideal drug for rapid recovery of consciousness after discontinuation (Mirenda and Broyles, 1995). In addition, accumulating evidence has shown that the occurrence of ICU delirium is a strong predictor of increased mortality and prolonged hospitalization (Ely et al., 2004). One potential mechanism for inducing delirium is the activation of the γ-aminobutyric acid receptor (Maldonado, 2008). Propofol and midazolam are γ-aminobutyric agonists, and both drugs have the potential to cause delirium in patients (Levine, 1994). However, due to the rapid metabolic distribution of propofol, the delirium induced by propofol may be not long-lasting and harmful as midazolam. In addition, previous studies have reported that dexmedetomidine and propofol have similar sedative effects, and there is no difference in the prognostic effects of dexmedetomidine and propofol on patients with MV. However, given that dexmedetomidine appears to be associated with a higher risk of adverse effects, such as hypotension, bradycardia and other cardiopulmonary complications (Anger et al., 2010; Jakob et al., 2012), propofol is more recommended in this study when choosing a sedation regimen.

This study reviewed the effects of statins on patients receiving MV therapy in ICU. Our results showed that there was no difference in mortality and the incidence of VAP between patients who took statins during MV and patients who did not receive statins, and the use of statins did not reduce mortality in patients with VAP. A meta-analysis reported by Yu SS mentioned that the use of statins before admission will benefit critically ill patients and can significantly reduce the duration of mechanical ventilation and short-term mortality (Yu et al., 2021b). However, Laurent’s study was against the view that statins improve the prognosis of VAP patients. The results showed that the 28-day mortality rate of VAP patients with simvastatin was 22.6% (95% CI, 15.7–31.5%), while Placebo was 14.3% (95% CI, 8.9–22.2%) (*p* = 0.06) (Papazian et al., 2013b). Previous studies had suggested that there was a significant difference in the prognosis of patients with a history of statin prescriptions compared with patients with those who urgently use statins. This anti-inflammatory effect of statins may need sufficient dose and treatment time (Brealey et al., 2011; Bruyere et al., 2014). Because our study did not have access to a patient’s prescription history of statins, inability to distinguish whether a patient is on temporary or long-term medication. we hope that this point can be fully taken into account in follow-up studies.

By comparing the prognosis of MV patients treated with and without insulin, we found that the use of insulin did not affect mortality of MV patients, the incidence of VAP or the mortality of VAP patients. However, in previous studies, MV patients who were intensively treated with insulin appeared to have a better prognosis, with possible mechanisms including the elimination of glucose-induced osmotic diuresis, enhancement of erythropoiesis, prevention of acute renal failure, maintenance of macrophage and neutrophil function, reduction of cholestasis, promotion of direct anabolic effects of respiratory muscles, and protection of patients the central and peripheral nervous systems. A study by Krinsley et al. showed that the mortality rate of critically ill patients who use insulin to control blood glucose has decreased by 29.3% (*p* = 0.02), and the length of stay in ICU has been reduced by 10.8% (*p* = 0.01) (Krinsley, 2004). Another prospective observational study performed a multivariate Logistic regression analysis of data on patients undergoing MV and found that the degree of blood glucose control was associated with risk of death and organ system dysfunction, and independent of insulin dose (Van den Berghe et al., 2003). Using insulin to control the blood glucose concentration to less than 100–110 mg/dl appears to provide the best benefit to the patient (Lewis et al., 2004). In this study, wedistinguished the specific conditions of insulin use in patients, but did not dynamically monitor the blood glucose concentration of the patients. Therefore, there is uncertainty whether it is the actual insulin dose received per se or the degree of euglycemia achieved that is responsible for the beneficial effects of intensive glycemic management. We hope that more literature in the future to assess the clinical and economic effects of insulin on ICU patients.

Our research has the following limitations. First, due to database limitations, we could not know the exact time of VAP onset, so we could not build a competing risk model. In this regard, we include VAP onset in cox regression and found that VAP occurrence did not elevate mortality risk. In addition, we also supplemented the subgroup analysis of mortality risk in VAP patients, the results of which did not differ significantly from those of MV patients, we think the results are stable. Secondly, our data mainly comes from the MIMIC database, so the risk of losing confusing data is real, such as the dynamic changes of gastric pH in patients using SUP and the history of statins in MV patients. Thirdly, our study also analyzes the combination of drugs, but the specific situation of the combination of drugs is not clear, and it is not possible to determine whether patients were on combination or alternate use, so we have not interpreted the results of this piece of the study in detail. Finally, this study only investigated the effect of drugs on the mortality of patients with MV, the incidence of VAP and the prognosis of patients with VAP, but did not explain the adverse effects of the drugs. Therefore, further high-quality original research or more scientific research is needed to draw a clear conclusion.

## Conclusion

We conducted this retrospective analysis to explore the effects of different drugs in patients with MV. Results showed that propofol was superior to midazolam in reducing in-hospital mortality in patients requiring mechanical ventilation. Further high-quality original research or a more scientific approach is required to draw definitive conclusions.

## Data Availability

The original contributions presented in the study are included in the article/Supplementary Material, further inquiries can be directed to the corresponding authors.

## Abbreviations

MV, mechanical ventilation; VAP, ventilator-associated pneumonia; SAPS II, simplified acute physiology score II; CCI, Charlson comorbidity Index; PPI, proton pump inhibitors; H2RA, Histamine2-receptor antagonist; MRSA, methicillin resistant Staphylococcus aureus; aOR, adjusted odds ration; aHR, Adjusted Hazard Ratio.
