# Supplementary material for: Effect of Stress Ulcers Prophylaxis, Sedative and Statin on Ventilator-Associated Pneumonia: A Retrospective Analysis Based on MIMIC Database
Source: Front Pharmacol. 2022 Jun 20;13:921422. doi: 10.3389/fphar.2022.921422 (PMC9251373; doi:10.3389/fphar.2022.921422)
Supplement: Supplementary file 1 [file Table1.docx]

**Supplementary Table 1: Univariate analysis of the effect of drugs on the death of MV patients.**

|  | **Survive (N=3363)** | **In-hospital death (N=1914)** | ***p*-value** |
| --- | --- | --- | --- |
| **Age** |  |  |  |
| Median [min, max] | 63.1 [18.0, 97.8] | 68.7 [18.1, 98.7] | **<0.001** |
| **Gender** |  |  |  |
| M | 1960 (58.3%) | 1058 (55.3%) | **0.036** |
| F | 1403 (41.7%) | 856 (44.7%) |  |
| **Ethnicity** |  |  |  |
| American Indian/Alaska native | 10 (0.3%) | 4 (0.2%) | 0.841 |
| Asian | 98 (2.9%) | 58 (3.0%) |  |
| Black/African American | 390 (11.6%) | 190 (9.9%) |  |
| Hispanic/Latino | 119 (3.5%) | 64 (3.3%) |  |
| White | 2082 (61.9%) | 1097 (57.3%) |  |
| Missing | 664 (19.7%) | 501 (26.2%) |  |
| **SAPS II** |  |  |  |
| Median [min, max] | 40.0 [6.00, 105] | 48.0 [7.00, 107] | **<0.001** |
| **SOFA** |  |  |  |
| Median [min, max] | 9.00 [0, 21.0] | 11.0 [0, 23.0] | **<0.001** |
| **CCI** |  |  |  |
| Median [min, max] | 5.00 [0, 16.0] | 7.00 [0, 19.0] | **<0.001** |
| **Diagnoses** |  |  |  |
| Respiratory | 558 (16.6%) | 236 (12.3%) | **<0.001** |
| CNS | 96 (2.9%) | 32 (1.7%) |  |
| Liver | 41 (1.2%) | 40 (2.1%) |  |
| Renal | 31 (0.9%) | 22 (1.1%) |  |
| Diabetes | 19 (0.6%) | 7 (0.4%) |  |
| Trauma | 203 (6.0%) | 108 (5.6%) |  |
| Other | 1940 (57.7%) | 1182 (61.8%) |  |
| Missing | 475 (14.1%) | 287 (15.0%) |  |
| **Acinetobacter baumannii** |  |  |  |
| N | 3319 (98.7%) | 1892 (98.9%) | 0.711 |
| Y | 44 (1.3%) | 22 (1.1%) |  |
| **Pseudomonas aeruginosa** |  |  |  |
| N | 3084 (91.7%) | 1785 (93.3%) | **0.048** |
| Y | 279 (8.3%) | 129 (6.7%) |  |
| **Klebsiella pneumoniae** |  |  |  |
| N | 3178 (94.5%) | 1797 (93.9%) | 0.391 |
| Y | 185 (5.5%) | 117 (6.1%) |  |
| **Escherichia coli** |  |  |  |
| N | 3077 (91.5%) | 1760 (92.0%) | 0.598 |
| Y | 286 (8.5%) | 154 (8.0%) |  |
| **MRSA** |  |  |  |
| N | 3163 (94.1%) | 1793 (93.7%) | 0.626 |
| Y | 200 (5.9%) | 121 (6.3%) |  |
| **Stenotrophomonas maltophilia** |  |  |  |
| N | 3279 (97.5%) | 1846 (96.4%) | **0.034** |
| Y | 84 (2.5%) | 68 (3.6%) |  |
| **WBC** |  |  |  |
| Median [min, max] | 9.00 [0.200, 208] | 12.9 [0.100, 206] | **<0.001** |
| Missing | 2 (0.1%) | 4 (0.2%) |  |
| **INR** |  |  |  |
| Median [min, max] | 1.20 [0.800, 8.30] | 1.40 [0.800, 27.4] | **<0.001** |
| Missing | 45 (1.3%) | 19 (1.0%) |  |
| **Lactate** |  |  |  |
| Median [min, max] | 1.40 [0.400, 19.6] | 1.80 [0, 28.2] | **<0.001** |
| Missing | 186 (5.5%) | 57 (3.0%) |  |
| **SUP** |  |  |  |
| PPI | 511 (15.2%) | 333 (17.4%) | **0.004** |
| H2RA | 500 (14.9%) | 281 (14.7%) |  |
| PPI or Sucralfate | 13 (0.4%) | 12 (0.6%) |  |
| H2 or Sucralfate | 0 (0%) | 2 (0.1%) |  |
| PPI or H2RA | 141 (4.2%) | 130 (6.8%) |  |
| PPI, H2RA or Sucralfate | 1 (0.0%) | 0 (0%) |  |
| Missing | 2197 (65.3%) | 1156 (60.4%) |  |
| **Sedative** |  |  |  |
| Propofol | 447 (13.3%) | 308 (16.1%) | **<0.001** |
| N | 2261 (67.2%) | 1223 (63.9%) |  |
| Dexmedetomidine | 22 (0.7%) | 11 (0.6%) |  |
| Midazolam | 38 (1.1%) | 48 (2.5%) |  |
| Dexmedetomidine or Propofol | 300 (8.9%) | 121 (6.3%) |  |
| Midazolam or Propofol | 158 (4.7%) | 135 (7.1%) |  |
| Dexmedetomidine, Midazolam or Propofol | 137 (4.1%) | 68 (3.6%) |  |
| **Statin** |  |  |  |
| N | 2977 (88.5%) | 1654 (86.4%) | **0.028** |
| Y | 386 (11.5%) | 260 (13.6%) |  |
| **Insulin** |  |  |  |
| N | 2646 (78.7%) | 1399 (73.1%) | **<0.001** |
| Y | 717 (21.3%) | 515 (26.9%) |  |
| **Antibiotic** |  |  |  |
| N | 192 (5.7%) | 89 (4.7%) | **<0.001** |
| Single antibiotic | 413 (12.3%) | 138 (7.2%) |  |
| Combined antibiotics | 2758 (82.0%) | 1687 (88.1%) |  |
| **Vasopressor** |  |  |  |
| N | 2666 (79.3%) | 1027 (53.7%) | **<0.001** |
| Y | 697 (20.7%) | 887 (46.3%) |  |
| **Length of Ventilation (day)** |  |  |  |
| Median [min, max] | 3.67 [2.00, 85.3] | 4.42 [2.00, 45.4] | **<0.001** |
| **VAP** |  |  |  |
| N | 2789 (82.9%) | 1662 (86.8%) | **<0.001** |
| Y | 574 (17.1%) | 252 (13.2%) |  |
| **Length of ICU stays (day)** |  |  |  |
| Median [min, max] | 9.13 [2.19, 99.6] | 8.04 [2.10, 61.0] | **<0.001** |
