# Supplementary material for: Effect of Stress Ulcers Prophylaxis, Sedative and Statin on Ventilator-Associated Pneumonia: A Retrospective Analysis Based on MIMIC Database
Source: Front Pharmacol. 2022 Jun 20;13:921422. doi: 10.3389/fphar.2022.921422 (PMC9251373; doi:10.3389/fphar.2022.921422)
Supplement: Supplementary file 2 [file Table2.docx]

**Supplementary Table 2: Univariate analysis of the effect of drugs on death in patients with VAP.**

|  | **Survive (N=574)** | **In-hospital death (N=252)** | ***p*-value** |
| --- | --- | --- | --- |
| **Age** |  |  |  |
| Mean (SD) | 60.9 (16.8) | 68.5 (13.9) | **<0.001** |
| Median [min, max] | 62.7 [19.6, 94.8] | 69.6 [24.1, 96.9] |  |
| **Gender** |  |  |  |
| Male | 361 (62.9%) | 151 (59.9%) | 0.464 |
| Female | 213 (37.1%) | 101 (40.1%) |  |
| **Ethnicity** |  |  |  |
| American Indian/Alaska native | 2 (0.3%) | 0 (0%) | 0.304 |
| Asian | 16 (2.8%) | 8 (3.2%) |  |
| Black/African American | 71 (12.4%) | 18 (7.1%) |  |
| Hispanic/Latino | 21 (3.7%) | 6 (2.4%) |  |
| White | 337 (58.7%) | 144 (57.1%) |  |
| Missing | 127 (22.1%) | 76 (30.2%) |  |
| **SAPS II** |  |  |  |
| Mean (SD) | 41.5 (15.6) | 47.4 (14.7) | **<0.001** |
| Median [min, max] | 40.0 [6.00, 92.0] | 47.0 [14.0, 107] |  |
| **SOFA** |  |  |  |
| Mean (SD) | 8.94 (4.13) | 10.0 (4.00) | **<0.001** |
| Median [min, max] | 9.00 [0, 21.0] | 10.0 [1.00, 21.0] |  |
| **CCI** |  |  |  |
| Mean (SD) | 5.27 (2.86) | 6.88 (2.70) | **<0.001** |
| Median [min, max] | 5.00 [0, 15.0] | 7.00 [0, 15.0] |  |
| **Diagnoses** |  |  |  |
| Respiratory | 97 (16.9%) | 29 (11.5%) | **0.011** |
| CNS | 16 (2.8%) | 4 (1.6%) |  |
| Liver | 3 (0.5%) | 7 (2.8%) |  |
| Renal | 5 (0.9%) | 2 (0.8%) |  |
| Diabetes | 4 (0.7%) | 0 (0%) |  |
| Trauma | 33 (5.7%) | 24 (9.5%) |  |
| Other | 327 (57.0%) | 141 (56.0%) |  |
| Missing | 89 (15.5%) | 45 (17.9%) |  |
| **Acinetobacter baumannii** |  |  |  |
| N | 559 (97.4%) | 240 (95.2%) | 0.166 |
| Y | 15 (2.6%) | 12 (4.8%) |  |
| **Pseudomonas aeruginosa** |  |  |  |
| N | 482 (84.0%) | 219 (86.9%) | 0.328 |
| Y | 92 (16.0%) | 33 (13.1%) |  |
| **Klebsiella pneumoniae** |  |  |  |
| N | 519 (90.4%) | 222 (88.1%) | 0.375 |
| Y | 55 (9.6%) | 30 (11.9%) |  |
| **Escherichia coli** |  |  |  |
| N | 513 (89.4%) | 226 (89.7%) | 0.992 |
| Y | 61 (10.6%) | 26 (10.3%) |  |
| **MRSA** |  |  |  |
| N | 535 (93.2%) | 234 (92.9%) | 0.974 |
| Y | 39 (6.8%) | 18 (7.1%) |  |
| **Stenotrophomonas maltophilia** |  |  |  |
| N | 543 (94.6%) | 234 (92.9%) | 0.415 |
| Y | 31 (5.4%) | 18 (7.1%) |  |
| **WBC** |  |  |  |
| Mean (SD) | 9.57 (3.89) | 15.4 (9.55) | **<0.001** |
| Median [min, max] | 9.00 [1.10, 26.6] | 13.2 [0.100, 54.5] |  |
| **INR** |  |  |  |
| Mean (SD) | 1.45 (0.592) | 1.69 (1.83) | **0.003** |
| Median [min, max] | 1.20 [0.900, 4.70] | 1.30 [0.900, 27.4] |  |
| Missing | 3 (0.5%) | 1 (0.4%) |  |
| **Lactate** |  |  |  |
| Mean (SD) | 1.98 (1.91) | 2.80 (3.20) | **<0.001** |
| Median [min, max] | 1.40 [0.400, 19.6] | 1.50 [0.300, 21.6] |  |
| Missing | 28 (4.9%) | 5 (2.0%) |  |
| **SUP** |  |  |  |
| PPI | 90 (15.7%) | 40 (15.9%) | 0.244 |
| H2RA | 92 (16.0%) | 43 (17.1%) |  |
| PPI or Sucralfate | 3 (0.5%) | 2 (0.8%) |  |
| PPI or H2RA | 38 (6.6%) | 30 (11.9%) |  |
| Missing | 351 (61.1%) | 137 (54.4%) |  |
| **Sedative** |  |  |  |
| Propofol | 70 (12.2%) | 36 (14.3%) | 0.279 |
| N | 358 (62.4%) | 149 (59.1%) |  |
| Dexmedetomidine | 5 (0.9%) | 2 (0.8%) |  |
| Midazolam | 12 (2.1%) | 7 (2.8%) |  |
| Dexmedetomidine or Propofol | 64 (11.1%) | 22 (8.7%) |  |
| Midazolam or Propofol | 25 (4.4%) | 21 (8.3%) |  |
| Dexmedetomidine, Midazolam or Propofol | 40 (7.0%) | 15 (6.0%) |  |
| **Statin** |  |  |  |
| N | 499 (86.9%) | 202 (80.2%) | **0.017** |
| Y | 75 (13.1%) | 50 (19.8%) |  |
| **Insulin** |  |  |  |
| N | 437 (76.1%) | 162 (64.3%) | **<0.001** |
| Y | 137 (23.9%) | 90 (35.7%) |  |
| **Antibiotic** |  |  |  |
| N | 17 (3.0%) | 1 (0.4%) | 0.052 |
| Single antibiotic | 26 (4.5%) | 9 (3.6%) |  |
| Combined antibiotics | 531 (92.5%) | 242 (96.0%) |  |
| **Vasopressor** |  |  |  |
| N | 452 (78.7%) | 144 (57.1%) | **<0.001** |
| Y | 122 (21.3%) | 108 (42.9%) |  |
| **Length of Ventilation (day)** |  |  |  |
| Mean (SD) | 7.09 (6.18) | 8.60 (6.62) | **0.002** |
| Median [min, max] | 5.15 [2.00, 52.5] | 6.77 [2.00, 45.4] |  |
| **Length of ICU stays (day)** |  |  |  |
| Mean (SD) | 15.8 (9.49) | 15.0 (8.88) | 0.287 |
| Median [min, max] | 13.8 [2.53, 79.0] | 13.0 [2.33, 60.8] |  |
